# Can single disease payment system based on clinical pathway reduce hospitalization costs in rural area? A case study of uterine leiomyoma in Anhui, China

**DOI:** 10.1186/s12913-018-3807-1

**Published:** 2018-12-20

**Authors:** Jing Peng, Mengran Zhang, Pingfeng Yu, Nan Wang

**Affiliations:** 1 Management School of Hefei University of Technology, Hefei, 230000 Anhui China; 2 School of Health Service Management, Anhui Medical University, Hefei, 230000 Anhui China

**Keywords:** Single disease payment, Clinic pathway, Uterine leiomyoma, Hospitalization expenditure, Rural China

## Abstract

**Background:**

Single disease payment program based on clinical pathway (CP-based SDP) plays an increasingly important role in reducing health expenditure in china and there is a clear need to explore the scheme from different perspectives. This study aimed at evaluating the effect of the scheme in rural county public hospitals within Anhui, a typical province of China,using uterine leiomyoma as an example.

**Methods:**

The study data were extracted from the data platform of the New Rural Cooperative Medical Office of Anhui Province using stratified-random sampling. Means, constituent ratios and coefficients of variations were calculated and/or compared between control versus experiment groups and between different years.

**Results:**

The total hospitalization expenditure (per-time) dropped from 919.08 ± 274.92 USD to 834.91 ± 225.29 USD and length of hospital stay reduced from 9.96 ± 2.39 days to 8.83 ± 1.95 days(*P* < 0.01), after CP-based SDP had implemented. The yearly total hospitalization expenditure manifested an atypical U-shaped trend. Medicine expense, nursing expense, assay cost and treatment cost reduced; while the fee of operation and examination increased (*P* < 0.05). The expense constituent ratios of medicine, assay and treatment decreased with the medicine expense dropped the most (by 4.4%). The expense constituent ratios of materials, ward, operation, examination and anesthetic increased,with the examination fee elevated the most (by 3.9%).The coefficient of variation(CVs) of treatment cost declined the most (− 0.360); while the CV of materials expense increased the most (0.186).

**Conclusion:**

There existed huge discrepancies in inpatient care for uterine leiomyoma patients. Implementation of CP-based SDP can help not only in controlling hospitalization costs of uterine leiomyoma in county-level hospitals but also in standardizing the diagnosis and treatment procedures.

## Background

“To see a doctor is expensive and difficult” has become a widespread saying in China. The problem may be attributed to two main problems of the Chinese health care system. First, with incomplete insurance coverage for urban and rural dwellers, issues such as uneven access, mixed quality of health care, increasing costs, and risk of catastrophic health expenditures have become the main discontent among the population. [1, 2] Second, with the distorted incentive mechanism, health care providers and hospitals were motivated by profits and incentivized to prescribe excessive and high-tech diagnostic tests and expensive pharmaceuticals to earn profits. In 2011, pharmaceutical expenditure accounted for 43% of total health expenditures [3], nearly tripled the Organization for Economic Cooperation and Development’s (OCED) average of 16% [4]. It is very important to resolve how to control the ever-increasing medical expenditure and promote reasonable development of medical institutions, especially in rural areas where equitable access to health care need to be increased and additional financial protections for vulnerable populations are needed [5]. Recently China government has piloted a bunch of new ways to respond to these issues, including the change of health insurance payment system and treatment guidelines that assure improved quality and emphasize prevention and primary care of chronic disease. Expenditure on hospitalization comprises one of the largest shares of total health-care spending in all countries [6–9] and inpatient services have typically been paid though single disease payment system (SDP) which is the focus of this study. More specifically, we tried to answer the following question: does SDP based on clinical pathway affect health care expenditures in rural area in China?

By definition, SDP classifies cases according to the principal diagnoses and the presence of co-morbidities and complications. Cases classified as having the same principal diagnoses without serious co-morbidities and complications will have the same payment criteria,which in most areas in China is used as maximum limit of the payment price not as the unified payment price. In china, SDP is often referred to as Diagnosis-related Groups (DRGs), yet they are not the same. DRGs classify cases according to the following variables: principal and secondary diagnoses, patient age and sex, the presence of co-morbidities and complications and the procedures performed [10], cases within the same DRG are economically and medically similar [8, 11]. Strictly speaking, single disease is an adjust form of DRGs [12], and some study also shows that countries that import an existing variant of a DRG-based system should be mindful of the need for adaptation [10]. So, the single payment system is easier to be implemented considering the current situation in China, especially in rural areas where the material and human resources are limited. The two core design characteristics of SDP are: (i) Disease identification and coding, which is adherent to International Statistical Classification of Diseases and Related Health Problems (ICD), although WHO has updated it to the 11th edition (ICD-11), the current disease classification in China is mainly based on ICD-10 and ICD-9; (ii) the payment criteria, which is based on the historical payment data or clinical pathway and the charging standards of medical service. SDP has gradually been put into trial in some provinces in China since the 1990s [13]. In 2017, the central government of china have issued related policies to combine SDP with clinical pathway (CP) in order to increase or maintain quality of medical services [14]. As pointed out by Yu Buqing et al., SDP can reduce induced consumption caused by fee-for-service and increase efficiency [15]. Researches by Liu Hongxiang and Hong Liu both suggest that SDP generally helps in decreasing hospital cost but also has adverse effects on medical service quality [16, 17]. Xu Wei et al. [18] commented that, SDP basically can contain the hospitalization costs and make the fee structure becoming more reasonable; the fixed payment played a role in standardizing the medical service behavior but decreased the actual compensation for patients. Hao-miao Li et al. [19] used Weiyuan county, Gansu as a sample and evaluated the effect of payment reform and found that the medical economic burden of inpatients diseased substantially after SDP was implemented. Although the length of hospital stay decreased, the economic burden on those not paid by SDP increased.

Based on these previous findings/observations, the general aim of our study is to expand the analysis of the effects of SDP based on clinical pathway, to see what would the effect of the two combined mechanisms be like and how is the outcome can contribute to the current existing problems in rural areas. Uterine leiomyoma is a very common disease for women. It affects up to 70% of women of reproductive age [20],and contributes to nearly 70% of all uterine sarcomas and a significant proportion of uterine cancer deaths. Its morbidity in China increased from 3.4% in 1970s to 5.2% in 2012 [21]. This research investigates cost control effect of patients with uterine leiomyoma before and after the implementation of CP-based SDP at county-level hospitals in Anhui, a typical agricultural province in China with a population of about 70 million. The per capita GDP in Anhui was 6417.8 USD(2017) which is similar to the per capita GDP level of many developing countries and regions,such as South Africa,Thailand and so on. [22]. Therefore, the main contribution of this paper is to compile experiences and to explore the design and implementation issues that rural areas in China face. Ultimately it will provide some ideas for policy-makers in other developing countries about how to control the health expenditure for people live in rural areas.

## Method

### Study population

Selection of study subjects proceeded in three steps. Step 1 divided Anhui Province into 3 regions (i.e., south, middle and north Anhui) according mainly to geographic locations, population density and economic status. Step 2 randomly selected 4, 4 and 3 counties from the 3 regions respectively and treated the county hospital within each of the counties as the site hospital. Here, the reason why only 3 rather than 4 counties were drawn from the north was because this is the most populated area in Anhui and hence the scale of the county-level hospitals is substantially larger than that in other areas. Step 3 searched for all eligible inpatients from the 11 county-level hospitals in the counties derived via step 2. Here, the eligibility criteria were inpatients who had: a) been admitted during 2010 through to 2015 with a principal diagnose as uterine leiomyoma (ICD10:D25) and b) received total /sub-total abdominal hysterectomy (ICD9CM-3:68.39/68.49). The patients who did not meet the diagnostic criteria or suffered serious complications were excluded. Finally, a total of 1432 inpatients were selected from the 11 hospitals.

### Data extraction

The study data were extracted from the data platform of New Rural Cooperative Medical Office of Anhui Province. Specific content of data extraction included: a) principal diagnosis (i.e., uterine leiomyoma); b) socio-demographics (age, sex, education); c) costs (on total hospitalization and on specific categories including medicine expense, nursing expense, ward fee, operation fee, examination fee, assay cost, treatment cost, anesthetic fee and other fees).

### Statistical analysis

The data collected were cleaned using EXCEL software first and then analyzed using SPSS16.0. The analysis comprised calculation /comparison of: a) means and standard deviations of total hospitalization expenditures and length of hospital stay for control and experiment period and for different years (Panel data technique); b) constituent ratios of categorical expenses; and c) coefficients of variation (CVs) of the total and categorical expenditures. The constituent ratio of any specific categorical cost was calculated by taking the categorical cost as the numerator and the total hospitalization expenditure as the denominator. The control period was defined as 2010 to 2012 and the experiment, 2013 to 2015 since CP-based SDP was introduced in late 2012. The student t-test method of approximate normal distribution was used in the power test for most of the differences between the control and experimental groups in terms of duration of hospital stay, total hospitalization expenditure and various categorical costs, with two-sided test level of α = 0.05. But for the expense on ‘others’, wilcoxon rank sum test (α = 0.05) was used to compare the difference between the control and experimental groups since the standard deviation was greater than the mean value. The total and categorical expenditures from 2010 to 2014 were converted to comparable costs in 2015 by taking CPI as the adjustment basis in order to eliminate the influence of inflation.and the expenditures from 2010 to 2015 were converted from CNY into U.S.dollars by taking 2015 exchange rate (1 Dollar = 6.2284 Yuan) as the adjustment rate.

### Ethics, consent and permissions

The study protocol had been reviewed and approved by the Biomedical Ethics Committee of Anhui Medical University.

## Results

### Comparison of total hospital expenditure and length of hospital stay

**Fig. 1:**
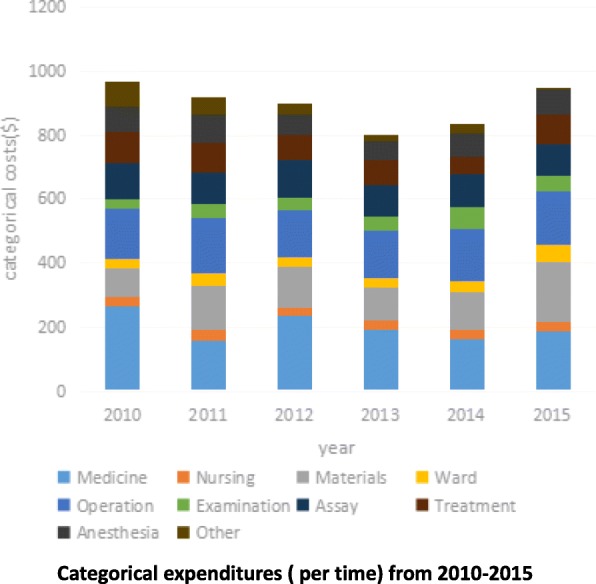
Categorical expenditures (per time) from 2010 to 2015

Table 1 shows the average hospitalization expenditures(per time) of uterine leiomyoma in experimental group and control group, which were 813.14 ± 221.81USD and 843.41 ± 253.77 USD respectively. Taking inflation (CPI) into account, the adjusted total expenditures were 834.91 ± 225.29USD and 919.08 ± 274.74USD respectively. The length of hospital stay of experimental group and control group was 8.83 ± 1.95 days and 9.96 ± 2.39 days. The differences in total hospitalization expenditure and duration of hospital stay between two groups were statistically significant (*P* < 0.01). Total hospitalization expenditure and length of hospital stay in experimental group are lower than that of the control group. The yearly total hospitalization expenditure manifested an atypical U-shaped trend (Fig. 1). More specifically, it started the highest (965.86 ± 299.06USD) in 2010, dropped year by year to its lowest (794.66 ± 206.12USD) in 2013, and then began to increase to a level (948.96 ± 563.89USD) below its start point. By comparison, the yearly length of hospital stay displayed a Z-shaped trend. It remained relatively high during the control period (before 2012) but dropped to a lower level in 2013 and remained at this level thereafter.

**Table 1:** Comparison of total hospital expenditure and length of stay (^−^x ± s)

Grouping and power test	Total hospital expenditure($)	Length of stay(day)
Control group		
2010	965.86 ± 299.06	9.777 ± 1.718
2011	915.93 ± 201.91	9.581 ± 2.407
2012	896.88 ± 281.15	10.185 ± 2.647
subtotal	919.08 ± 274.92	9.960 ± 2.390
Experimental group		
2013	794.66 ± 206.12	8.571 ± 1.826
2014	833.76 ± 191.09	8.861 ± 1.986
2015	948.96 ± 563.89	8.857 ± 1.590
subtotal	834.91 ± 225.29	8.830 ± 1.950
Power test (subtotal for control vs. experiment group)		
t	5.537	8.581
P	< 0.01	< 0.01

Noted:The total expenditures from 2010 to 2015 were converted from CNY to U.S. dollar by taking 2015 exchange rate (1 Dollar = 6.2284 Yuan) as the adjustment basis

### Comparison of categorical costs

Table 2 provides descriptive statistics about categorical and inflation-adjusted costs. The differences in materials expense, ward fee and anesthetic fee between the two groups had no statistical significance (*P* > 0.05); while the differences in medicine expense, operation fee, assay cost and treatment cost were statistically significant (*P* < 0.05). Medicine expense, nursing expense, assay cost and treatment cost were lower in the experimental group than in the control group; but operation fee and examination fee were higher in the experimental group than in the control group.

**Table 2:** Comparison of various costs (^−^x ± s)

Cost category	Medicine	Nursing	Materials	Ward	Operation	Examination	Assay	Treatment	Anesthetic	Other
Control group ($)										
2010	264.11± 206.22	33.65± 15.09	134.72± 36.48	29.78± 9.53	155.50± 44.03	32.34± 16.16	112.99± 46.75	94.71± 104.95	82.21± 17.31	74.52± 47.89
2011	155.71± 95.70	35.02± 21.49	136.36± 51.33	40.88± 16.26	167.60± 44.80	45.70± 34.89	99.14± 29.17	92.60± 68.50	87.18± 22.83	55.75 ± 58.97
2012	235.13± 151.44	27.58± 16.04	123.88± 63.01	30.62± 13.76	149.69± 46.24	36.30± 23.54	119.96± 45.96	76.85± 65.99	63.69± 21.68	33.17 ± 33.78
Subtotal	228.48± 164.41	30.60± 17.24	115.97± 57.87	32.28± 13.88	154.55± 45.82	36.95± 24.78	114.29± 44.24	84.56± 79.28	73.03± 23.22	48.45± 46.83
Experimental group ($)										
2013	187.47± 99.31	32.29± 17.72	97.93± 70.65	30.21± 10.14	151.15± 23.36	40.98± 24.50	95.24± 43.87	77.38± 44.84	59.98± 13.98	22.04± 36.80
2014	161.89± 92.54	27.80± 14.18	121.99± 74.26	33.11± 14.04	163.10± 39.83	68.28± 40.41	100.30± 32.04	55.80± 29.40	72.74± 26.53	27.73± 37.99
2015	189.16± 163.29	25.18± 10.43	189.91± 185.60	52.69± 21.64	164.50± 45.36	52.36± 30.75	99.00± 75.23	91.45± 58.82	77.34± 33.72	7.37± 4.52
Subtotal	166.08± 97.94	28.18± 14.52	122.56± 83.96	33.73± 14.74	161.82± 38.73	65.29± 39.60	99.67± 36.56	59.95± 34.68	71.53± 26.12	26.11± 37.18
t	7.305	2.517	−1.660	−1.743	−2.848	−15.839	5.959	6.193	1.055	8.687
P	< 0.01	0.012	0.097	0.082	< 0.01	< 0.01	< 0.01	< 0.01	0.292	< 0.01

Noted:The categorical expenditures from 2010 to 2015 were converted from CNY to U.S. dollar by taking 2015 exchange rate (1 Dollar = 6.2284 Yuan) as the adjustment basis

### Comparison of cost constituent ratio

Table 3 gives comparisons of cost constituent ratios between the experimental group and control group. The difference in constituent ratio of nursing fee had no statistical significance (*P* = 0.35); while the other fees (*P* < 0.05) had statistical significance. The constituent ratios of medicine expense, assay fee and treatment cost were lower in the experimental group than in the control group. Of these decreasing ratios, the ratio of medicine expense dropped the most (by 4.4%), followed by treatment cost (1.6%). Looking at the increasing ratios, those of materials expense, ward fee, operation fee, examination fee and anesthetic fee were higher in the experimental group than in the control group and among them, the ratio of examination fee elevated the most (by 3.9%), followed by operation fee (2.8%). In terms of yearly trend (Fig. 1), the expenses on medicine, nursing and others manifested continuous decreases from 2010 through to 2015; while expenses on the remaining categories mimicked the atypical U-shaped trend of total hospitalization cost describe above.

**Table 3:** Comparison of cost constituent ratio (^−^x ± s)

Cost category	Medicine	Nursing	Materials	Ward	Operation	Examination	Assay	Treatment	Anesthetic	Other
Experimental group	0.192 ± 0.086	0.036 ± 0.029	0.139 ± 0.067	0.043 ± 0.019	0.204 ± 0.057	0.080 ± 0.043	0.122 ± 0.039	0.073 ± 0.035	0.088 ± 0.029	0.033 ± 0.043
Control group	0.236 ± 0.114	0.035 ± 0.020	0.128 ± 0.052	0.037 ± 0.017	0.176 ± 0.054	0.05 ± 0.026	0.130 ± 0.048	0.089 ± 0.056	0.085 ± 0.027	0.055 ± 0.055
Difference value	−0.041	0.001	0.011	0.006	0.026	0.037	−0.006	−0.018	0.005	−0.023
t	7.402	−0.955	−3.454	−5.668	−8.841	−21.079	2.614	5.648	−2.795	7.81
P	< 0.01	0.35	< 0.01	< 0.01	< 0.01	< 0.01	< 0.01	< 0.01	< 0.01	< 0.01

### Comparison of coefficient of variation

**Fig. 2:**
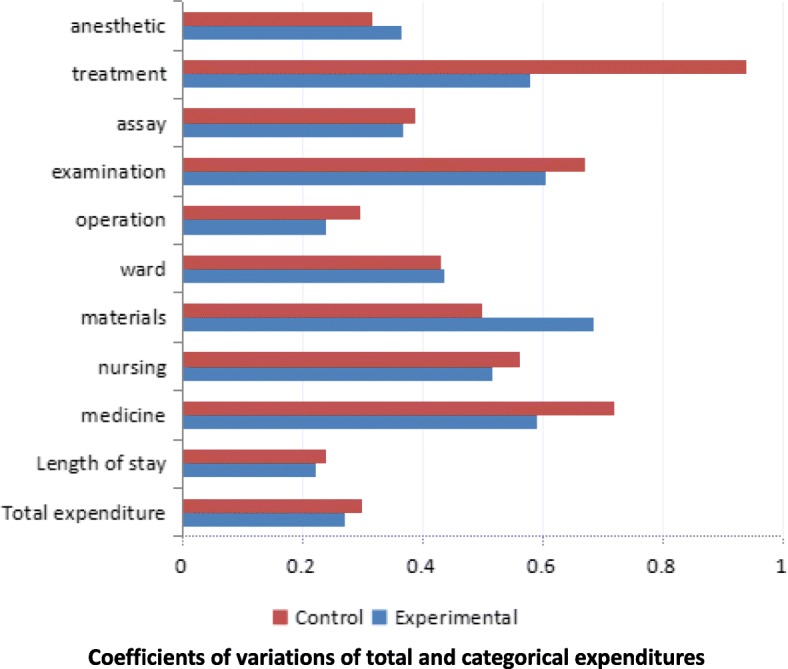
Coefficients of variations of total and categorical expenditures

Figure 2 present the CVs of all the cost variables studies. As compared with the control group, the CV of total hospitalization expenditure, length of hospital stay, medicine expense, nursing expense, operation fee, examination fee and treatment cost reduced in the experiment group, with the CV of treatment cost declined the most (− 0.360), followed by CV of medicine expense (− 0.130). Three of the CVs increased including that of material expense, ward fee and anesthetic fee. Of these increased CVs, the CV of materials expense increased the most (0.186) and the change in the CV of ward fee was the smallest (0.008). Looking at the figures within experimental group, the CV of materials fee was the largest (0.685), followed by that of examination (0.606), medicine (0.590) and treatment fee (0.579).The CV of length of stay was the smallest (0.221), followed by that of operation fee (0.239) and total hospitalization expenditure (0.270).

## Discussion

### Reduction in total hospitalization expenditure and length of hospital stay

Our analysis revealed that average total hospitalization expenditure (per time) of the experimental group was 524.25 Yuan(84.17 USD) lower than that of the control group, and average duration of hospital stay shortened 1.13 days to 8.83 days. Wang Ying et al. found that after implementation of clinical pathway, total hospitalization expenditure reduced 840.70 Yuan(134.98 USD)on average, and 698.34 Yuan(112.12 USD)at least [23]. The effect of CP-based SDP on total hospitalization expenditure and duration of hospital stay for uterine leiomyoma in county-level hospitals from our study was lower than that of CP-without SDP. This difference may be explained mainly by the fact that SDP sets limits on expenditures but CP provides reference for use of necessary diagnosis and treatment procedures and each procedure inevitably incurs expenditures. In other words, the effect of CP-based SDP reflects the trade-offs between cost control and practice of important procedures. This observation is supported by the atypical U-shaped yearly trend shown in Table 1. China government announced its New Health Reform Plan in 2009,which clearly stated that China will provide “safe, effective, convenient, and affordable” health services to all urban and rural residents [24]. Then starting from 2010, a series of new policies have been enacted including SDP, zero-markup for medicines, capped proportion of revenue from medicines [25, 26]. All of policies have cost-containing effect. Anhui has been the champion province of this new reform. So, it is reasonable to witness year by year drop in total hospitalization expenditures on uterine leiomyoma. Then the implementation of CP-based SDP, in 2013, reversed the dropping trend since the CP clearly stipulates standard diagnosis and treatment procedures. The Z-shaped yearly trend observed with length of hospital stay may be caused by the fact that the aforementioned cost-containing measures do not set specific limits on duration of hospitalization; while CP specifies diagnosis and treatment procedures on a daily base and have clear implications or standards on length of stay. Research by Marguerite L et al. shows that, the mean length of hospital stay of benign uterine Leiomyoma is 2.3 days in the USA [27].Although the duration of hospital stay in county-level hospitals dropped to 8.83 days, it still has significant gaps with worldwide level, so some measures are needed to further decrease the length of hospital stay.

### Changes in categorical costs and their constituent ratio

Although the total hospitalization cost decreased after implementation of CP-based SDP, the changes in costs on specific categories were mixed with some decreased, others increased, and still others remained relatively stable. The decrease in medicine, treatment and assay costs and other fees may be attributable to the joint effects of CP-based SDP and other health reform measures as mentioned earlier. Of these four categories, the medicine expenses witnessed an apparently greater reduction than the remaining three. This may be explained mainly by the fact that in addition to CP-based SDP and other commonly applicable policies, Anhui had enacted policies (e.g., zero-markup and capped revenue proportion) which restricted use of medicine in particular. The increases in expenses on examination and operation may be explained partly by changed requirements that favor more examination and operation procedures due to the CP. In addition, examination and operation- related procedures may also have been used, by some of the hospitals and doctors, as alternative means for coming up the revenue losses due to enactment of the zero-markup policy and others..The relatively stable costs on ward, materials and anesthetic may due mainly to their relatively fixed nature.

### Large coefficient of variation of costs

Our study revealed large CVs of category specific as well as total expenses (from 0.221 to 0.938). These variations may have been caused by two domains of factors, i.e., disease heterogeneity and decision discrepancy. The former refers to different patients with same uterine leiomyoma diagnosis but different related health conditions that require different diagnosis and treatment procedures; while the latter concerns patients with a same health condition but adopted different diagnosis and treatment procedures due to differences in decision-making. By comparison, treatment, medicine and examination are much more complex in nature than operation and length of stay. This explains why the CVs of expenses on treatment, medicine and examination were much greater than that on operation and length of stay. Our analysis uncovered mixed changes in the CVs of expenditures on different categories of inpatient service. Nine out of the 11 CVs studied witnessed various degrees of decreases after introduction of CP-based SDP. This suggests that CP helped in reducing practice discrepancies at least to some extent. It is hard to say whether these CV reductions are enough or not given the data from this study. Theoretically, clinical pathway management should aim at restricting decisional discrepancies while leaving adequate room for disease heterogeneity. Otherwise, it ranks the risk of over-standardization and therefore hinders personalized and quality care. Another point worth noting relates to the CVs of expenditures on materials and operation. These two CVs showed increases rather than decreases. Again, reasons underlining this reverse trend need to study. It may because, in part, that the CP provides or encourages more operation and material alternatives. It may also due partly to that the CP has more vague specifications to operations and materials than to treatment and others.and thus clinicians felt more freedom in choosing materials and operation methods.

### Limitations and intentions

The study also has limitations. First, the subjects of our study came from county-level hospitals within a single province in China. Readers are well cautioned about potential location biases, though the social and economic conditions in Anhui Province are similar to the majority of provinces in the country. Second, there enacted significant reform policies other than CP-based SDP during this research period such as the essential drug policy, and drug zero-markup policy. These also had effects on the hospitalization cost, especially the medicine fee. Interpretation of the research findings needs to be most careful. Third, the research data were extracted from past medical records preserved by 11 county level hospitals. Data completeness and quality confined this study to most preliminary analysis. For example, the costs studied included only direct healthcare cost without any consideration of non-healthcare and indirect costs. Encouraged and informed by findings from this study, we are going to do a second phase evaluation of CP-based SDP which uses: a) nationwide samples; b) prospectively recorded data in addition to medical records; c) descriptive as well as economic modeling.

## Conclusions

There existed huge discrepancies in inpatient care for uterine leiomyoma patients. Implementation of CP-based SDP was beneficial. It helped not only in controlling hospitalization costs of uterine leiomyoma in county-level hospitals but also in standardizing the diagnosis and treatment procedures. As a new scheme, CP-based SDP merits further policy and research efforts. In developing CP-based SDP, the primary focus should be put on the most important and complex procedures like treatment, medicine, examination. At the same time, adequate attention should also be paid to seemingly simple or non-significant procedures so as to reach a balanced mechanism which not only ensures standardizing major procedures and cost categories but also prevents potential unwanted “bypassing” effects.
